# Investigating Cancerous Exosomes’ Effects on CD8+ T-Cell IL-2 Production in a 3D Unidirectional Flow Bioreactor Using 3D Printed, RGD-Functionalized PLLA Scaffolds

**DOI:** 10.3390/jfb13010030

**Published:** 2022-03-11

**Authors:** Daniel Karami, Akhil Srivastava, Rajagopal Ramesh, Vassilios I. Sikavitsas

**Affiliations:** 1 School of Chemical, Biological, and Materials Engineering, University of Oklahoma, Norman, OK 73019, USA; daniel.s.karami@gmail.com; 2 Stephenson School of Biomedical Engineering, University of Oklahoma, Norman, OK 73019, USA; 3 Department of Pathology, The University of Oklahoma Health Sciences Center, Oklahoma City, OK 73104, USA; akhil-srivastava@ouhsc.edu (A.S.); rajagopal-ramesh@ouhsc.edu (R.R.); 4 Stephenson Cancer Center, University of Oklahoma Health Sciences Center, Oklahoma City, OK 73104, USA

**Keywords:** exosome, bioreactor, cancer, surface modification, fluid flow

## Abstract

Exosomes from cancer cells are implicated in cancer progression and metastasis, carrying immunosuppressive factors that limit the antitumor abilities of immune cells. The development of a real-time, 3D cell/scaffold construct flow perfusion system has been explored as a novel tool in the study of T-cells and exosomes from cancer cells. Exosomes from human lung cancer (H1299 and A549) cells were co-cultured in a unidirectional flow bioreactor with CD8+ T-cells immobilized onto 3D-printed RGD-functionalized poly(L-lactic) acid (PLLA) scaffolds and assessed for IL-2 production. The IL-2 production was investigated for a wide range of T-cell to exosome ratios. With the successful incorporation of the RGD binding motif onto the PLLA surface at controllable densities, CD8+ T-cells were successfully attached onto 2D disks and 3D printed porous PLLA scaffolds. T-cell attachment increased with increasing RGD surface density. The diameter of the attached T-cells was 7.2 ± 0.2 µm for RGD densities below 0.5 nmoles/mm^2^ but dropped to 5.1 ± 0.3 µm when the RGD density was 2 nmoles/mm^2^ due to overcrowding. The higher the number of cancer exosomes, the less the IL-2 production by the surface-attached T-cells. In 2D disks, the IL-2 production was silenced for T-cell to exosome ratios higher than 1:10 in static conditions. IL-2 production silencing in static 3D porous scaffolds required ratios higher than 1:20. The incorporation of flow resulted in moderate to significant T-cell detachment. The portions of T-cells retained on the 3D scaffolds after exposure for 4 h to 0.15 or 1.5 mL/min of perfusion flow were 89 ± 11% and 30 ± 8%, respectively. On 3D scaffolds and in the presence of flow at 0.15 ml/min, both H1299 and A549 cancerous exosomes significantly suppressed IL-2 production for T-cell to exosome ratios of 1:1000. The much higher level of exosomes needed to silence the IL-2 production from T-cells cultured under unidirectional flow, compared to static conditions, denotes the importance of the culturing conditions and the hydrodynamic environment, on the interactions between CD8+ T-cells and cancer exosomes.

## 1. Introduction

Exosomes are small, 30–100 nm in diameter vesicles that are exuded by every cell in the body under normal conditions and are involved in intracellular communication and housekeeping functions. However, in cancer, exosomes have been implicated in cancer-related processes and drug resistance [1]. Although it has been 30 years since the discovery of exosomes, the exosome field is still in its infancy, and the exact roles of exosomes in tumor progression and metastasis are still unclear due to their submicron size and the complexity of their interactions [2]. Both the contents—which include biomolecules such as tumor suppressing and transcriptional proteins; ribonucleic acids (RNA), such as micro-RNA and non-coding RNA; deoxyribonucleic acids (DNA) [3,4]; and lipids [5,6,7,8]—and the rates of dispersion of exosomes exuded from cancer cells have been shown to differ.

Breast cancer cells, when compared to a normal mammary epithelial cell line, exuded exosomes at an almost two orders of magnitude higher rate. Several other studies have revealed similar findings showing increased exosome release In vitro and in vivo in animals and humans [9,10,11]. Exosomes released from cancer cells, unlike normal cells, exude RNA-induced silencing complex, or RISC, complex-associated mRNA, and this complex is essential to targeting and silencing genes [12]. Indications have appeared that the exosomal secretion rates and exosomal uptake rates are increased in cancer patients when compared to healthy patients [13,14,15], implying that the tumor microenvironment influences them significantly [10].

Multiple studies have shown that cancer cell exosomes impact and modulate chemosensitivity and drug resistance by transferring chemoresistance to nearby recipient cells by transport of bioactive molecules such as RNAs [16,17,18]. Innate multi-drug resistances, or MDR, are commonplace in cancer, since cancer cells overexpress drug efflux pumps, transporters, and resistance proteins; and acquired drug resistance is tied to signaling pathways triggered by the tumor microenvironment, which is heavily populated by exosomes [19]. Given the central role of exosomes in cell–cell communication, they are linked to the resistance of cancers to drugs, but not only in intuitive ways, such as gene transfer or microenvironment signaling [20,21,22]. As exosomes impact the tumor microenvironment with their transfer of bioactive molecules and drug resistances, they inevitably interact with the immune system. Both the innate immune system and adaptive immune system function are hostile to the tumor microenvironment and have roles that naturally hinder tumorigenesis [23,24,25]. Cancer cells primarily interact with the cytotoxic T-lymphocytes (CD8+) and natural killer (NK) cells [26]. The regulatory role of exosomes has recently appeared in the literature: their regulation of the synapse formation between antigen-presenting cells (APC) and T-cells, promoting a response that may play a role in inhibiting immunosurveillance [27,28,29].

As nucleated cells express molecules for MHC Class I, their respective exosomes do as well, because of the surface antigens obtained from the parent cell membrane, such that they are able to interact with the cytotoxic lymphocytes and NK cells [30]. Cancer-related antigens are contained in tumor-derived exosomes that may initiate an immune response, and multiple studies [31,32,33] suggested that tumor-secreted exosomes can also become antigens for interactions with CD8+ T-cells indirectly through APC presentation and cross-dressing [32,34,35,36]. These factors are once again tied to the bioactive carriers within exosomes which have also been shown to impact NK cell immunity [37] and work by transferring micro-RNA to recipient cells [38,39]. Such deactivation can be linked to the inactivation of the T-cells, resulting in the downregulation of interleukin-2 (IL-2) or TNF-α cytokine production, or upregulation of IL-6 [40]. In order to understand the effects of exosomes in cancer, it is important to study the interactions between exosomes and T-cells in settings that better mimic the in vivo microenvironment where such interactions occur naturally. When preparing exosome T-cell co-cultures In vitro in suspension cultures, several inconsistencies between the in vivo microenvironments and the specific cell culture approach can be identified, implying that In vitro studies require more attention [41,42,43,44,45].

In vitro models create not only an ideal starting point for medical and biological research, but also an important subset, complementing the more complex in vivo models. Results of In vitro testing should be compared with and verify more complex tests, especially in vivo models and testing [46], including in studies involving cancer [47]. While small animal models more precisely showcase the complexity of the cancer microenvironment, discerning, visualizing, and extracting data are not only challenging and difficult, but expensive as well [47,48]. The complexity of In vitro cancer models of tumors varies, and they range from 2D monocultures to 3D multicellular structures and microenvironments [49]. They have provided insights on growth, proliferation, migration, and drug effectiveness [50,51,52]. In vitro systems can be flexibly tailor-made to reduce in vivo variability. Synthetic polymers can utilize proteins and peptides which enhance cellular proliferation due to elevated cell adhesion and ECM development [53,54,55]. Cancer cells and immunological cells exhibit weaker cell adhesion properties and fewer bioactive sites [56,57], which can be alleviated with common surface moieties for adhesion, such as cadherins [58] and peptide motifs [59]. The easy implementation of a wide variety of cancer cell lines in In vitro culture systems has made them attractive in biological studies [60,61], as they match some of characteristics of tumors [62,63], but certain discrepancies remain, necessitating In vitro models to reach better agreement with in vivo studies.

Fluid flow and shear rates provide beneficial dynamic environments for cellular cultures and modeling, and are showcased in the oncological field with microscale systems. Microfluidics have been commonplace in the oncological community for its modeling, based on microscale control of laminar flow for nutrient and drug transport [64,65], cell characteristics, pharmaceutical properties, and interactions [66,67]. 3D constructs, such as immobilized hydrogels, have been used to investigate microfluidic and microscale dynamics for tumor models [68,69], but diffusion cannot be controlled as closely with natural scaffolds when compared to porous scaffolds based on synthetic polymers [70]. Macroscale flow-perfusion bioreactors could be an appropriate next step to more accurately modeling tumor progression, since tumorigenesis is a delicate balance of both macroenvironmental and microenvironmental aspects [71], wherein the functional connections between cancer cells and their neighboring microenvironments can result in tumorigenesis [72] and phenotypic alterations [73]. Increased cell viability and nutrient availability have been observed in macroscopic systems, and the volume densities varied as much as five times between scales [74,75]. When compared to static 3D cultures, flow perfusion has been shown to increase cell proliferation and cell homogeneity within tissue-like structures and exhibit morphology and phenotypes resembling those of xenografts [76]. Biomechanical stimulation due to shear stresses has been often overlooked as a means of tumor progression [77,78], and 3D macroscale systems present a controlled In vitro environment to explore these dynamic conditions.

In this study, we tested the immunosuppressive nature of two types of cancerous human exosomes on immobilized CD8+ human T-cells in a dynamic 3D In vitro flow perfusion bioreactor. Activated immune systems typically seen in cancer and infectious diseases seem to be immunosuppressed in many types of cancer. To evaluate this interplay among immune cells, cancer cells, and their respective exosomes, it is experimentally advantageous to immobilize one of the active players. Immobilization of CD8+ T-cells would best isolate interactions with circulating factors and can be achieved with the utilization of surface modification and attachment using peptides on polymeric scaffolds to mediate testing variability. A model that encapsulates the complex nature of the cancer and immunological macroenvironments and microenvironments would set a foundation for future testing and validation. The goals of this study were to (1) generate RGD-functionalized PLLA scaffolds with controllable RGD densities, (2) explore the ability of RGD-modified PLLA scaffolds to encourage the attachment of T-cells, (3) investigate if T-cells can be cultured on 3D PLLA polymeric surfaces in a unidirectional flow bioreactor, and (4) study the effect of different levels of exosomes from lung cancer cells on the activity of T-cells residing on the surfaces of 3D PLLA polymeric scaffolds.

## 2. Materials and Methods

### 2.1. 2D PLLA Disks’ Preparation

#### 2.1.1. Material

2D poly(L-lactic) acid (PLLA) disks were utilized due to their availability, high reproducibility, and low production costs, along with the ability of the raw PLLA to generate 3-dimensional porous scaffolds in a reproducible way. The 2D disks were prepared in accordance with procedures that have been previously published [79,80]. PLLA (NatureWorks LLC; grade 6251D; 1.4% D-enantiomer, MW = 108,500 D) pellets were used to produce the 2D disks.

#### 2.1.2. Production

Approximately 2.5 g of PLLA pellets were weighed and then combined with 35 mL of chloroform. The pellets were mixed with a stir bar on a magnetic mixer for approximately 45 min, completely dissolving them. The heterogeneous mixture was then poured evenly into three 50 mm petri disks. The containers were set overnight at room temperature to evaporate. After the evaporation step, 2D disks of 50 mm diameter were collected. The 50 mm disks were then stamped using a fixed stencil into 8 mm disks. Each new disk was sterilized using 95% ethanol, allowed to sit in sterile PBS to leech off ethanol for 1 h, and then stored into a vacuum chamber. Disks were used for cell seeding within 24 h from their sterilization.

### 2.2. 3D PLLA Porous Scaffold Preparation

#### 2.2.1. Material

The method used to generate 3-dimensional porous PLLA scaffolds was 3D printing. In order to feed the polymer into the 3D printer, PLLA had to be in the form of a filament. A specialized extruder (Filastruder, Snellville, GA, USA) was used to produce this filament. PLLA pellets were placed in the hopper of the extruder to heat up, and then extruded into a 1 mm diameter filament. The filament was subsequently used in a 3D printer and, similarly to the PLLA pellets, was vacuum stored after preparation. Prior to usage, 3D scaffolds were placed in a biosafety cabinet and purged of air with a syringe vacuum method in PBS, cleaned with 95% ethanol, and then allowed to sit in sterile PBS to leech off ethanol for 1 h.

#### 2.2.2. Printing

**Figure 1:**
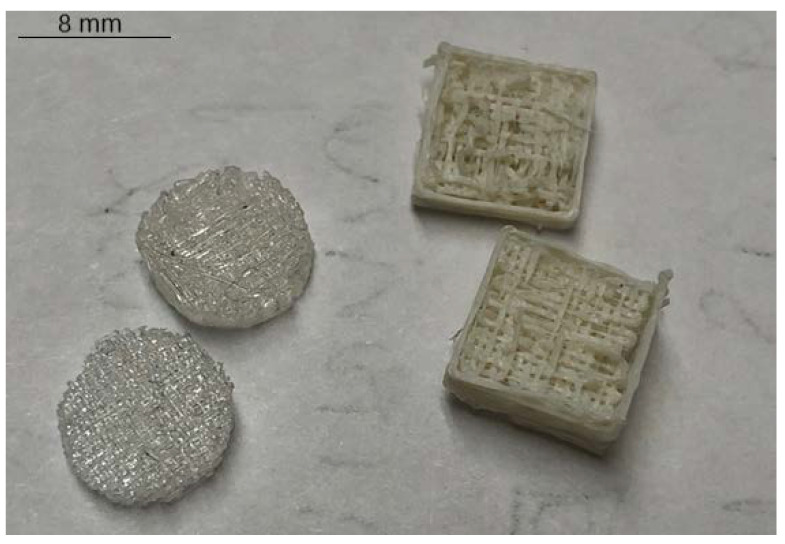
3D scaffolds made with a prototype commercial filament, and pre-stamped scaffolds using a custom extruded PLLA filament.

The 3D printer used was the MakerBot^®^ Replicator^®^ Fifth Generation Desktop 3D Printer. The filament was placed into the extruder and heated to 195 °F; then it was extruded to a 0.5 mm diameter at a rate of 0.5 cm/s. Scaffolds were printed in laddered extrusion patterns to produce square, 2 mm × 8 mm × 8 mm ones. 3D printed scaffolds, as seen in Figure 1, were then stamped using the same stencil as was used for the 2D disks.

### 2.3. Surface Modification

#### 2.3.1. 2D PLLA Disks

The protocol for the surface modification of the 2D disks was similar to previously published procedures [79,80]. 2D disks were prepared as above. Each 8 mm disk was immersed in a 70:30 acetone–water solution of poly-ε-Cbz-L-lysine (Sigma-Aldrich; Poly-ε-Cbz-L-lysine; MW = 500–4000 KD), which will now be referred to as Poly K, ranging from 1 mg/mL to 10^−7^ mg/mL. Disks were placed in a shaker for 12 h to promote the entrapment of the Poly K mixture on the PLLA surface before being removed to be washed with 3 cycles of Triton X-100 and sterile PBS.

Poly K’s presence on the disks was determined by a horseradish peroxidase (periodate-HRP) reaction with the amino acid chain. Disks modified with Poly K were reacted with 600 µL of 10^−8^ M of HRP ABTS ((2,2′-azinobis-(3-ethylbenzothiazoline-6-sulfonate)) Substrate Solution (Thermo Fisher; 1-Step™) for 2 h. The provided ABTS kit was used to determine HRP’s presence at 405 nm on a Synergy HT Multi-Mode Microplate Reader (Bio-Tek) with standards of measured Poly K prepared for calibrations. As specified by the manufacturer, H_2_O_2_ and ABTS reagent were dilated with citrate buffer and then incubated with disks in 600 µL of the solution. Poly K decay on 2D disks was tested by performing the HRP detection method on disks that remained in the biohood at room temperature for a period of up to 7 days. 

**Figure 2:**
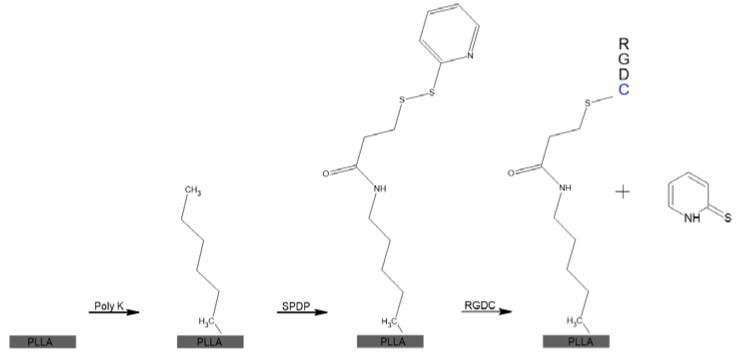
Breakdown of the surface modification, starting with PLLA and adding Poly K, SPDP, and finally RGDC [79,80].

With Poly K incorporated onto the surfaces of the disks of the PLLA, functionalization could occur to chemically bind, via amine coupling, RGD peptides to the Poly K that is entrapped onto the surface of the scaffold. Poly K modified disks were incubated with 600 µL of 1 mM succinimidyl 3-(2-pyridyldithio)propionate (SPDP; Thermo Fisher, Suwanee, GA, USA) in HEPES buffer for approximately 30 min. Similar washing cycles with Triton-X 100 and sterile PBS were used after the reaction. The disulfide bond present in SPDP was utilized with a disulfide reduction to place the RGDC onto the surfaces. SPDP-linked scaffolds were then incubated with 600 µL of 100 µM RGDC (AnaSpec; Cell Adhesive Peptide (RGDC)) for 1 h and rinsed as in the previous steps. A breakdown of the chemistry of the surface can be seen in Figure 2. The subsequently released pyridine-2-thiol can be seen at a wavelength of 343 nm and can be used to determine the concentration of reacted RGDC.

#### 2.3.2. 3D PLLA Porous Scaffolds

Scaffold porosity was found by comparing the ratio of the measured scaffold weight to the calculated scaffold volume. 3D scaffolds were modified following the same protocol as the 8 mm 2D disks by modifying the procedure of the entrapment of Poly K and incorporation of RGDC. The presence of the 3D porous network that needed to be accessed by the Poly K and RGDC moieties during their respective scaffold incorporations necessitated the use of vacuum pressure. 3D printed scaffolds were placed in glass vials with each concentration of Poly K, and then vacuumed repeatedly with a needle syringe. Subsequent steps using SPDP and RGDC were done using the same vacuum method, and each step was allowed to proceed for the same period as the one used in 2D disks. After modification, scaffolds were left in sterile PBS in a biosafety cabinet and used within 24 h.

#### 2.3.3. RGD Surface Density

The RGD surface density was calculated using an indirect method by producing a standard curve of the overall RGD bound onto the surface of the construct. The pyridine-2-thiol reaction can be used to approximate the number of total bonds formed, and from there an indirect measurement of the density of reacted RGD can be obtained. Known amounts of RGDC and SPDP were reacted to form a standard curve and disks were tested in sets of 9 to generate the average RGD reacting per Poly K entrapment concentration before scaling to the construct surface area.

### 2.4. T-Cell Culturing, Preparation, and Phalloidin Staining

Human CD8+ T-cells were obtained from StemCell Technologies (StemCell Technologies Vancouver, Canada; Human Peripheral Blood CD8+ T-cells, Frozen; 1 × 10^7^ cells per vial). As cells were labeled, profiled, and documented from the same donor designated by StemCell Technologies, T-cells were used directly from storage. T-cells were cultured throughout the studies in RPMI 1640 culture medium (Thermo Fisher, Suwanee, GA, USA) with 1% antibiotic-antimycotic and 10% fetal bovine serum. To determine the exact number of T-cells used, flow cytometry (BD Accuri C6, East Rutherford, NJ, USA) was used on the T-cell samples before use. An Alexa Fluor™ 488 Phalloidin (Fisher, Suwanee, GA, USA) kit was used to stain for phalloidin on the surface of each disk, per the manufacturer’s instructions.

### 2.5. Culture Methodology Set-Up

#### 2.5.1. Static 2D and 3D Set-Up

Static conditions for attachment, spreading, and exosome co-cultures were tested using 48-well plates. 2D PLLA disks were placed into their respective wells. Working volumes for T-cell attachment and exosome co-culture for static conditions were 1 mL. 3D static scaffolds also used the same set-up of a 48-well plate. 3D static scaffolds were placed into their respective wells, and 1 mL working volumes were used.

#### 2.5.2. Bioreactor Set-Up

**Figure 3:**
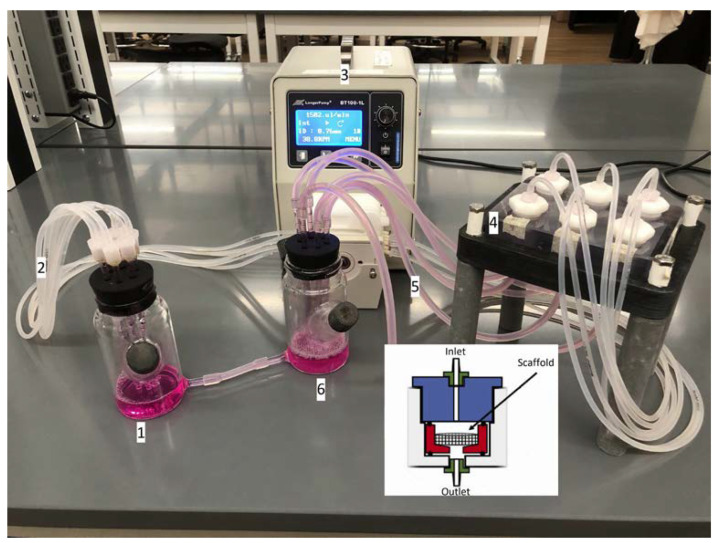
Bioreactor set-up for flow perfusion testing. A reservoir (1) container is linked with tubing (2) to a LongerPump BT100-1L pump (3), then to the insert of the scaffold cassettes (4), and finally, to the outlet tubing (5) leading to another container (6). The inset shows the schematic of the inside a chamber (all six are identical). The scaffold is form-pressed to assure that fluid flows through the porous structure and not around, with the inlet above and outlet below.

The bioreactor’s main body and cassettes were set up prior to use. The bioreactor system was sterilized three-days prior and allowed to dry before proceeding. The system was operated and purged of air in a biosafety cabinet, and then placed in a 37 °C incubator for 6 h equilibrate its temperature with the environment. Once equilibrated, prepared 3D scaffolds with attached T-cells were placed into each cassette in the biosafety cabinet. Different flow rates were used based using the peristaltic pump specifications. The flow rates used were 0.15 and 1.5 mL/min based on the pump limitations to test extreme flow values for our system. Each cassette was connected to its own vial of media and had a working volume of 5 mL of culture media. For flow rate testing, the bioreactor system was connected to a single reservoir to accommodate necessary working volumes for the system and can be seen in Figure 3.

### 2.6. PMA Preparation and Stimulation

#### 2.6.1. 2D PLLA Disks

2D 8 mm disks were carefully cleaned of previous culture media and prepared for cytokine stimulation. PMA (Sigma-Aldrich; 12-O-Tetradecanoylphorbol 13-acetate, St. Louis, MI, USA) was used to stimulate activated T-cells to produce IL-2. A 10 µM concentration of PMA in the culture medium was created and then reacted with the 2D disks for 4 h. To minimize potential contaminants, the samples were taken and centrifuged at 1000 RPM for 3 min (Allegra X-15R, Beckman Coulter, Florence, KY, USA) and the supernatant used for cytokine analysis.

#### 2.6.2. 3D Printed PLLA Scaffolds

Static 3D scaffolds were stimulated similarly to the 2D 8 mm disks. T-cells were cultured with the 3D printed RGD-modified scaffolds for 12 h to attach prior to flow testing. A 10 µM concentration of PMA was used on the 3D scaffolds and stimulated for 4 h. Samples were centrifuged and the supernatant used. 3D printed scaffolds used in the bioreactor system were run and stimulated with PMA. The collection process was similar to that for the static 3D scaffolds.

#### 2.6.3. Fluid Calculations

A simplified calculation to obtain order of magnitude approximation for the shear rate through the system was needed, as previous studies on our system have been done that closely examined the fluid mechanics [81]. Under Newtonian conditions, mean velocity, pore diameter, and media viscosity can be used to find the shear stress through the scaffolds [82]. The mean velocity can be calculated for the incoming fluid using:

(1) $$V_{m}=\frac{Q}{\phi \pi {\left(\frac{D}{2}\right)}^{2}}$$

where $V_{m}$ is mean velocity in cm/s, Q is the volumetric flow rate in mL/s, ϕ is the porosity of each scaffold, and *D* is the diameter of the scaffold. For a Newtonian fluid through a pipe, and assuming a parabolic fluid flow, the shear stress can be found:

(2) $$\gamma =\frac{8\mu V_{m}}{d}$$

where γ is the shear stress in dynes/cm^2^, μ is the media viscosity in g/cm∗s, $V_{m}$ is the mean velocity in cm/s, and *d* is the diameter of the pores in the scaffold in µm.

### 2.7. Exosome Characterization and Co-Culture

The H1299 and A549 human lung cancer cell lines were obtained from ATCC (Manassas, VA, USA). Confluent H1299 and A549 cultures were used to collect their conditioned media. Modified ultracentrifugation and differential high-speed methods were used to isolate H1299 and A549 exosomes [83]. Dead cells and debris were removed with centrifugation of 3000× *g* for 10 min and 10,000× *g* for 20 min (Beckman Coulter Avanti J-26S, Beckman Coulter Life Sciences, Indianapolis, IN, USA). The supernatant was filtered using a 0.22 µm Millex^®^ Syringe filter (EMD Millipore, Cork, Ireland) and the ultracentrifuge filtrate was centrifuged again at 100,000× *g* for 90 min. The exosome pellet was washed with PBS and stored at −80 °C. Exosomes were collected and purified from H1299 and A549 human lung cancer cell lines. Nanosight was used to identify and characterize the exosomes with capture settings of 696 slider shutter, 73 slider gain, 25.0 FPS, 1498 frames, and 22.2 °C. The co-culture procedure involved attaching T-cells to constructs exposed to exosomes that were part of the media flowing through the bioreactor.

#### 2.7.1. 2D PLLA Disks

Exosomes were diluted from their initial concentrations to 10^3^ exosomes/mL to be used in exosome ratios with T-cells. Ratios of 1:1, 1:5, 1:10, 1:20, and 1:100 T-cells to exosomes were used to test exosomes’ effects on T-cells. 2D disks were incubated concurrently with exosomes.

#### 2.7.2. 3D Printed PLLA Scaffolds

Static 3D scaffolds were cultured similarly to 2D 8 mm disks. However, for the flow perfusion bioreactor system, the T-cells to exosomes ratios were 1:10, 1:100, and 1:1000. Exosomes were incorporated into the culture media and circulated while testing under the lowest flow condition of 0.15 mL/min.

### 2.8. IL-2 Indirect ELISA

An IL-2 ELISA kit (Abcam; Human IL-2 ELISA Kit High Sensitivity, Cambridge, UK) was used to detect IL-2 production from PMA-stimulated T-cells. The assay was performed as per the manufacturer’s instructions. Samples and standards were prepared, and the assay wells were prepared in triplicate. The antibody linkers were placed into each well while the functionalization was underway. Washing buffer was used thrice after each subsequent step. After functionalizing, the dye buffer was used, and the results were recorded at 450 nm.

### 2.9. Statistics

Following data collection and analysis, a one-way ANOVA and Tukey tests (for multiple comparisons) were conducted to determine if there was a significant effect (*p*-value < 0.05). The number of samples per T-cell study was 3, whereas the surface modification studies related to RGD evaluation used *n* = 9.

## 3. Results

### 3.1. RGD Modification

**Figure 4:**
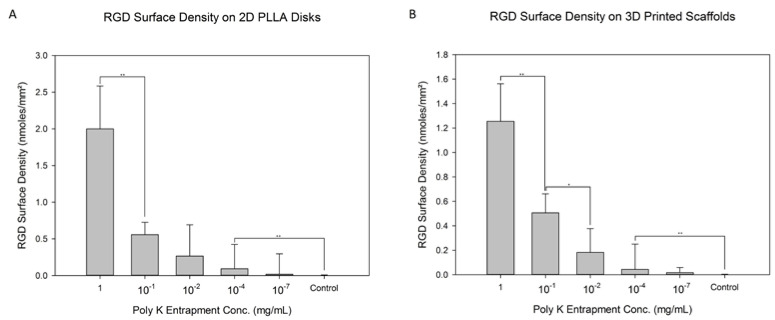
RGD surface density on (**A**) 2D disks and (**B**) 3D scaffolds. Poly K was entrapped onto the surface of the PLLA, was reacted with SPDP and then was functionalized with RGDC to present RGD on the surface. Statistics were calculated with Tukey tests, ** *p* < 0.01 and * *p* < 0.05.

RGD was attached to the Poly K surface via amine coupling, which allowed further modification to present the RGD peptide on the surface. Different Poly K entrapped surfaces were used, characterized by the level of Poly K in the solution during the partial solubilizing process with the acetone–water solution. Those levels varied from 1 mg/mL to 10^−7^ mg/mL Poly K. There was a control group with no Poly K. The resulting trendline is $\left(\frac{\mathrm{nmoles}}{mm^{2}}\right)=\frac{7.545*\left[Poly\;K\;Entrapment\;Concentraion\;in\;mg/mL\right]}{1+2.777*\left[Poly\;K\;Entrapment\;Concentration\;in\;mg/mL\right]}$ with an $R^{2}$ of 0.963. The RGD surface density was calculated by measuring the free pyridine-2-thiol from the SPDP and RGDC reaction and was used to measure the RGD functionalization on the scaffold. As seen in Figure 4A, there were significant differences between 1 and 10^−1^ mg/mL Poly K and 10^−4^ mg/mL Poly K and the control. The surface with the lowest level of Poly K did not show a statistically significant difference from the control surface. There does not seem to have been a significant difference between the control and the 10^−4^ and 10^−7^ mg/mL surfaces for the 2D RGD attachment. The RGD surface density ranged from 2.00 ± 0.58 nmoles/mm^2^ for 1 mg/mL to 0.02 ± 0.28 nmoles/mm^2^ for 10^−7^ mg/mL. The continuous increase in the RGD surface density with the Poly K entrapment solution does not seem to have reached saturation. Overall, the RGD surface density increased with Poly K entrapment concentration.

Figure 4B showcases the same RGD amine-coupling reaction shown in Figure 4A through 2D disks, but now on 3D scaffolds, where the porosity of the 3D printed scaffolds was found to be 85 ± 4%. The overall functionalization process was the same as that for the 2D disks, utilizing a functionalized PLLA surface that was modified with SPDP and RGDC. Similarly, the level of reacted RGD varied depending on Poly K entrapment from 1 mg/mL to 10^−7^ mg/mL (as reflected in the concentration in solution during entrapment), producing a similar trendline to that in Figure 4A of $\left(\frac{nmoles}{mm^{2}}\right)=\frac{8.3*\left[Poly\;K\;Entrapment\;Concentraion\;in\;mg/mL\right]}{1+5.636*\left[Poly\;K\;Entrapment\;Concentration\;in\;mg/mL\right]}$ with an $R^{2}$ of 0.987. A control without entrapped Poly K was included. There were significant differences between 1 and 10^−1^ mg/mL, 10^−1^ and 10^−2^, and 10^−4^ mg/mL and the control. Similarly to the 2D disks, the average RGD surface density is used to signify the Poly K entrapment and the subsequent RGD functionalization. Ultimately, there seems to have been a trend of increasing Poly K entrapment and functionalized RGD on the surface of the 3D scaffold, as observed for the 2D surfaces.

### 3.2. 2D T-Cell Attachment

**Figure 5:**
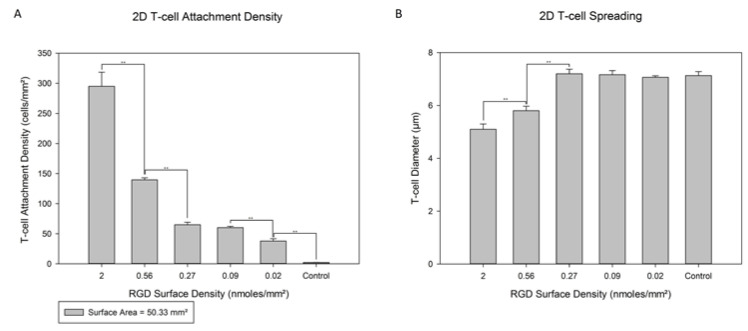
T-cells on (**A**) 2D disks with varying RGD surface densities and (**B**) cell diameters via a phalloidin stain. Disks were RGD-functionalized and then seeded with T-cells for 1-day before being stimulated with PMA for 4 h in static conditions. Statistics were calculated with Tukey tests, ** *p* < 0.01.

All RGD-functionalized disks having RGD surface densities between 2.00 ± 0.56 (1 mg/mL) and 0.02 ± 0.28 (10^−7^ mg/mL) nmoles/mm^2^ (including controls) were used in the T-cell attachment tests. Higher levels of RGD surface density resulted in higher numbers of T-cells being attached, as reported in Figure 5A. Significant differences in T-cell attachment were observed between 2D surfaces having surface densities of 2.0 ± 0.6 and 0.56 ± 0.17 nmoles/mm^2^, 0.56 ± 0.17 and 0.27 ± 0.43 nmoles/mm^2^, 0.09 ± 0.3 and 0.02 ± 0.3 nmoles/mm^2^, and 0.02 ± 0.3 nmoles/mm^2^ and none (control). The highest RGD surface density on 2D disks was 2.00 ± 0.56 nmoles/mm^2^ and yielded 295 ± 23 cells/mm^2^. Lowering the RGD surface density resulted in fewer T-cells on the surface, with the lowest density being 48 ± 2 cells/mm^2^ on disks with 0.02 ± 0.28 nmoles/mm^2^ RGD surface density. It needs to be noted that no T-cells attached to controlled surfaces. There was no significant difference in T-cell attachment between 0.27 ± 0.43 nmoles/mm^2^ and 0.09 ± 0.3 nmoles/mm^2^.

A phalloidin assay was performed to more clearly visualize the attached cells and to allow the measurement of the levels of their spread on the 2D disks with different RGD surface densities. The diameters are shown in Figure 5B. These diameters ranged from 7.2 ± 0.2 to 5.1 ± 0.3 µm, for RGD surface densities of 0.02 ± 0.28 and 2.00 ± 0.56 nmoles/mm^2^, respectively. Interestingly, the lowest spreading was observed in 2D disks having the highest RGD surface densities of 2.00 ± 0.56 nmoles/mm^2^ with a diameter of almost 5.2 ± 0.4 µm. Decreasing the RGD surface density to 0.27 ± 0.43 nmoles/mm^2^ increased the T-Cell spreading to a diameter of 7.2 ± 0.3 µm; and all discs with lower surface densities had an almost identical T-cell diameter to that. It needs to be denoted that for disks having RGD surface densities higher than 0.27 ± 0.43 nmoles/mm^2^, greater T-cell overcrowding was observed. At the highest RGD surface density tested, the disks appeared crowded with adhering T-cells.

### 3.3. 3D T-Cell Attachment

**Figure 6:**
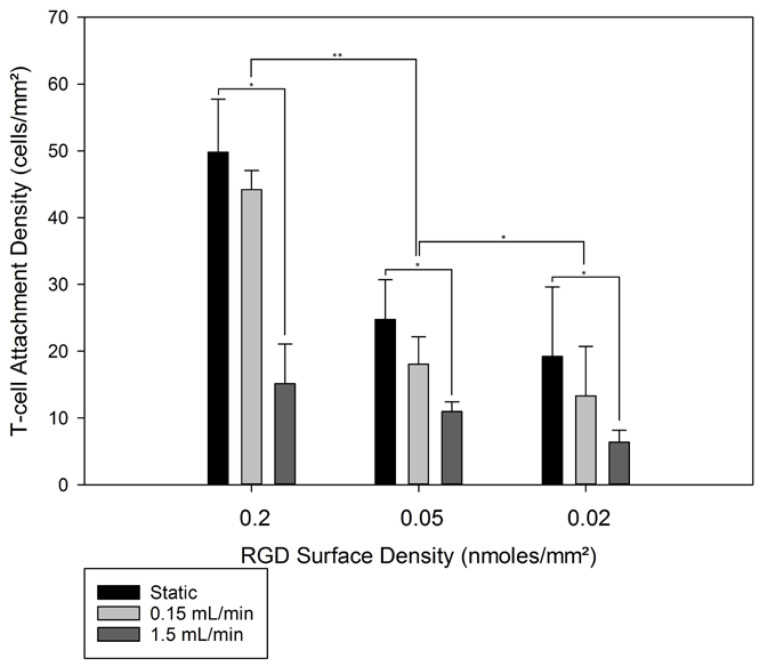
Incorporation of unidirectional bioreactor fluid flow with RGD-functionalized 3D scaffolds (and their attached T-cells) whose RGD surface densities ranging from 0.2 to 0.02 nmoles/mm^2^. 3D scaffolds were pre-seeded before entering the bioreactor and resisted flow for 4 h before PMA stimulation. Statistics were calculated with Tukey tests, ** *p* < 0.01 and * *p* < 0.05.

Figure 6A shows the attachment of T-cells on 3D porous scaffolds with various RGD functionalization densities. Following T-cell seeding, 3D scaffolds were cultured either under static conditions or in the presence of unidirectional flow. Two different flowrates were used when T-cell-loaded 3D scaffolds were exposed: 0.15 and 1.5 mL/min. The effective superficial velocity through each scaffold ranged from 1.2 ± 0.5 mm/min to 12.4 ± 0.1 mm/min. Since in 2D disk experiments, significant T-cell overcrowding was observed on disks with RGD surface densities higher than 0.27 ± 0.43 nmoles/mm^2^, 3D scaffolds with similar RGD surface densities were excluded from 3D T-cell attachment. The scaffolds excluded from 3D attachment studies were the ones with the two highest RGD surface densities, as reported in Figure 4B. Three different RGD surface densities were used to elucidate potential effects of the RGD surface densities on the behavior of the attached T-cells. Control groups with no RGD were used, as no T-cell attachment was possible on these constructs.

Static culturing resulted in T-cell densities as high as 50 ± 8 cells/mm^2^ for the RGD surface density of 0.27 ± 0.43 nmoles/mm^2^, all the way down to 13.3 ± 7.4 cells/mm^2^ for 0.02 ± 0.28 nmoles/mm^2^, as seen in Figure 6. The exposure of the seeded T-cells to continuous unidirectional fluid flow for 4 h resulted in a decrease in T-cells on the 3D scaffolds. That decrease was more pronounced at the high flow rate of 1.5 mL/min. A clear pattern was observed for each RGD surface density: no exposure to flow resulted in the highest T-cell numbers, and the high flow rate of 1.5 mL/min decreased T-cell attachment significantly. For the highest RGD surface density, the highest T-cell attachment density was observed at the RGD density of 0.2 nmoles/mm^2^ with 50 ± 8 adherent cells/mm^2^. Exposure of these scaffolds to 0.15 mL/min unidirectional flow allowed 89 ± 11% of the statically seeded T-cells to remain on the surface, whereas 1.5 mL/min unidirectional flow decreased the remaining adherent cell density to only 30 ± 8% of what was first present on that scaffold. Decreasing the RGD surface density from 0.2 to 0.05 nmoles/mm^2^ resulted in a drop in the observed T-cell surface density to 25 ± 6 cells/mm^2^. Further exposure of the scaffolds with 0.05 nmoles/mm^2^ to 0.15 or 1.5 mL/min resulted in 18 ± 4 and 11 ± 1 cells/mm^2^. For the lowest RGD surface density of 0.27 ± 0.43 nmoles/mm^2^, the static seeding resulted in drops in the T-cell attachment density to 69 ± 13% and 19 ± 12% for the 0.15 and 1.5 mL/min flow rates, respectively. The shear stress through each scaffold, with a pore diameter of 100 µm, was 0.047 ± 0.001 dynes/cm^2^ or 0.47 ± 0.001 dynes/cm^2^ for 0.15 and 1.5 mL/min, respectively, and can be considered low shear (<1 dyne/cm^2^ for T-cells) [84]. For all 3D RGD surface densities, the T-cell attachment density dropped by 20% ± 10% when the T-cells were exposed to a shear force of approximately 0.05 dynes/cm^2^, or decreased by 67% ± 18% (compared to the static controls) when the T-cells were exposed to a shear force of approximately 0.5 dynes/cm^2^ for 4 h. To allow the maximum number of T-cells to be present in the co-culture experiments adhering T-cells to exosomes, a unidirectional flow rate of 0.15 mL/min was used in the perfusion bioreactor.

### 3.4. Exosome Co-Culture

**Figure 7:**
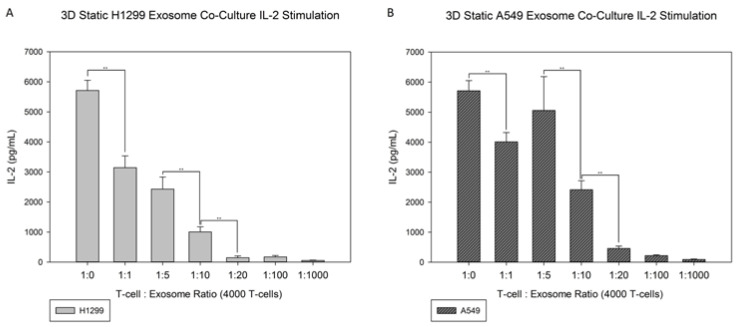
3D static exosome co-cultures of RGD-functionalized scaffolds with (**A**) H1299 and (**B**) A549 cancerous exosomes. The baseline IL-2 production is compared with production with T-cell to exosome ratios from 1:1 to 1:1000 before stimulation with PMA for 4 h. Statistics were calculated with Tukey tests, ** *p* < 0.01.

**Figure 8:**
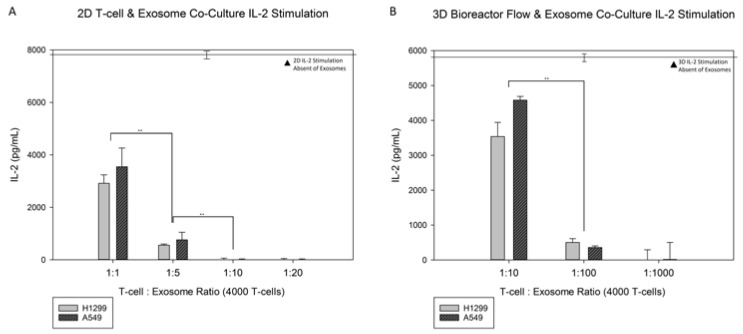
Comparison of IL-2 production in both RGD-functionalized (**A**) 2D disks of H1299 and A549 and (**B**) 3D scaffolds of H1299 and A549 with increasing T-cell to exosome ratios. Static 2D disks were co-cultured. 3D scaffolds were tested with unidirectional bioreactor fluid flow, with scaled fluid contents, before PMA stimulation for 4 h. Statistics were calculated with Tukey tests, ** *p* < 0.01.

Figure 7A,B showcases the results of the static exosome co-cultures with T-cells on 3D modified scaffolds, and these can be compared to the results in Figure 8A, which showcase the 2D disks co-cultured with H1299 and A549. The H1299 exosomes’ mean size was 167.0 ± 8.4 nm at a final concentration of 1.75 × 10^5^ particles/mL, and the A549 exosomes’ mean size was 161.3 ± 4.2 nm at a final concentration of 2.83 × 10^9^ particles/mL. The 3D static conditions for both H1299 and A549 exosome co-cultures present similar results to their modified 2D counterparts. Higher levels of exosomes resulted in lower IL-2 production from T-cells attached onto the RGD-modified disks. T-cell to exosome ratios of 1:10 and 1:20 resulted in no detectable IL-2 production, whereas at the 1:1 and 1:5 T-cell to exosome ratios, the IL-2 production was 2900 ± 300 or 600 ± 50 pg/mL for H1299-derived exosomes, and 3500 ± 700 or 800 ± 300 pg/mL for A549-derived exosomes, respectively. Significant differences can be seen between the cancerous exosomes. The difference from the no-exosomes baseline was 5700 ± 330 pg/mL for 1:1, 1:5 and 1:10 (H1299), and 1:10 and 1:20 (A549) T-cell to exosome ratios. The rate of IL-2 production (Figure 7A,B) decreased as the T-cell to exosome ratio increased in both cases. Very little IL-2 production occurred for the 1:20 T-cell to exosome ratios and higher. It needs to be noted that no statistical difference was observed between the 1:1 and 1:5 T-cell to exosome ratios with A549 cancerous exosomes.

Figure 8A shows the RGD-modified 2D PLLA disks individually co-cultured with exosomes from both H1299 and A549 cancerous cell lines. There was an observable difference in the IL-2 production of T-cells cultured on 2D PLLA disks without exosomes (7600 ± 390 pg/mL) and the IL-2 production of their exosome co-cultured counterparts. Exosomes from H1299 and A549 impacted the IL-2 production and resulted in T-cell deactivation, based on the lowered IL-2 production, as the ratio of exosomes to T-cells increased. There were significant differences between the baseline IL-2 production of T-cells on 3D scaffolds and the 1:10 T-cell to exosome ratios of 3500 ± 400 pg/mL and 4600 ± 500 pg/mL for H1299 and A549, respectively. The IL-2 production continued to decrease to 500 ± 100 pg/mL and 350 ± 50 pg/mL for H129 and A549, respectively, at a 1:100 T-cell to exosome ratio. No detectable IL-2 production was observed for either cancerous exosome co-culture at a 1:1000 T-cell to exosome ratio.

## 4. Discussion

We knew the development of an experimental model that allows CD8+ T-cells to be exposed to a flowing phase of cancer-exuded exosomes would be beneficial towards our understanding of the interactions between immune cells and cancer exosomes. To accomplish this goal, we (1) created RGD-functionalized scaffolds with controllable RGD densities, (2) confirmed the ability of RGD-modified scaffolds to bind CD8+ T-cells, (3) verified that attached T-cells can be cultured in the presence of unidirectional fluid flow, and (4) identified the effective cancer exosome to T-cell ratio to suppress IL-2 production in both 2D disk cultures and 3D static and 3D flow perfusion cultures.

Controllable RGD densities were achieved on RGD-functionalized polymeric disks and scaffolds. In both 2D disks and their 3D counterparts, RGD surface densities increased with Poly K entrapment concentrations; however, these differences were only significant at high levels of Poly K entrapment and RGD modification. Similar trends were observed in previous studies where the same technique was used to incorporate RGD onto PLLA disks and scaffolds [80]. For the same Poly K entrapment concentrations, the RGD densities on the 2D disks and the 3D scaffolds were comparable. However, at the highest level of Poly K entrapment concentration, the level of RGD density on the 3D scaffolds was 40% lower than that accomplished on the 2D disks. That discrepancy can be attributed to the presence of diffusional limitations when Poly K entrapment is taking place inside the porous network of the 3D scaffolds.

The functionalized PLLA disks were successful in achieving T-cell attachment using the commonplace binding motif of RGD. The T-cells not only attached onto the surface of RGD-modified disks, but their cell density was directly correlated with the RGD surface density as well. The T-cells attached to the modified RGD surface produced IL-2 at detectable levels. The IL-2 production was proportional to the T-cell density established on the 2D disks. A similar response was observed previously with T-cells attached on RGD-functionalized PEG hydrogels [85]. The absence of the specific RGD densities on the PEG-RGDS hydrogels prevents a direct comparison of the efficiency of T-cell binding between the two studies. It can be noted that in the absence of RGD functionalization, both PEG hydrogels and PLA do not support T-cell attachment, so it can be deduced that in both studies the T-cell attachment achieved post RGD functionalization was due to the presence of the RGD motif.

An important observation during analysis of T-cell attachment on 2D modified disks is the overcrowding effect at high RGD densities. The T-cells were densely packed, in continuous contact, and were forming a monolayer. Such overcrowding resulted in a significant decrease in the diameters of the attached T-cells that became more pronounced at the highest level of RGD surface density. On the other hand, in the absence of overcrowding, which happens when the RGD surface density was below 0.56 ± 0.17 nmoles/mm^2^, the RGD density did not seem to affect the diameter of the attached T-cells. This behavior contrasts with the increased mesenchymal stem cell spreading on increased RGD-functionalized PLLA surfaces. At the same time, it is in line with the utilization of integrins during their trans-endothelial and interstitial migration [86]. It is also in line with the ability of T-cells in vivo to squeeze through diameters smaller than those of average T-cells based on physiological conditions when responding to immune signals [87], providing an explanation for our observations at high RGD surface densities. As overcrowding appears to have a significant effect on T-cells, RGD surface densities above 0.27 ± 0.43 nmoles/mm^2^ were not used in the subsequent studies involving T-cell resistance to flow and interactions with exosomes.

Static 3D cultures were first tested to determine the viability of T-cell attachment onto a porous, 3D printed, and RGD-functionalized, scaffold, and to determine the starting T-cell attachment density prior to testing under flow conditions. In order to be able to conduct experiments where T-cells are exposed to exosomes that are incorporated in the perfused media, the T-cells must withstand the shear forces that will be generated by the unidirectional flow. Higher flow rates result in higher T-cell detachment. At the highest flow rate, the level of detachment was 67% ± 18%, denoting that the approximated shear force of 0.04 dynes/cm^2^ is near the critical value that results in T-cell detachment. At the lowest flow rate, where the average shear was 0.007 dynes/cm^2^, the level of detachment was not significant, implying that T-cells can withstand that lower shear force when bound to the RGD. It needs to be noted here that the shear forces just represent order of magnitude values, as the actual shear force profiles inside 3-dimensional scaffolds are complex, following distributions that are controlled by porosity, pore size, and flow rate, among others [88]. These findings provide important insights into the mechanobiology of T-cells and their interactions with RGD [89]. Interestingly, RGD surface density did not seem to significantly impact T-cell detachment under different flow conditions. Very high RGD densities that resulted in T-cell congestion and lower diameters were excluded from the perfusion experiments, potentially preventing a correlation between detachment and RGD density from being identified.

Under static culture conditions, even at the 1:1 T-cell to exosome ratio, there was significant silencing of T-cells cultured statically on 2D disks (compared to the baseline shown in Figure 8A). The lowest level of exosomes that resulted in almost complete absence of IL-2 production was the 1:10 T-cells to exosome ratio. It should be noted that the 2D disks were cultured for 4-h under static conditions, which resulted in the continuous presence of added exosomes in the immediate vicinity of the T-cells. T-cell silencing in the presence of cancerous exosomes has been reported extensively in the literature with the T-cells being in suspension cultures [90,91]. Regarding IL-2 production by T-cells in T-cell silencing, our findings confirm the observations made in suspension cultures in the context of cultures where T-cells are attached to an RGD-functionalized surface.

When exosomes were added to T-cells attached to RGD-functionalized 3D scaffolds, the suppression of IL-2 production was moderate compared to T-cells co-cultured on 2D disks. While on 2D disks, 1:10 T-cell to exosome ratio resulted in the absence of IL-2 production. The same outcome was accomplished by a 1:20 T-cell to exosome ratio in 3D scaffolds. The presence of T-cells in the interior of the porous scaffold network necessitated the efficient diffusion of exosomes for the exosome T-cell interaction to take place. Diffusional limitations on the mobile exosomes may provide an explanation for the requirement of higher levels of exosomes needed to achieve the silencing of IL-2 production from the T-cells in 3D scaffolds. It could also explain the moderate silencing of IL-2 production at lower T-cell to exosome ratios, 1:1 and 1:5, in 3D cultures. Overall, greater effectiveness of H1299 derived exosomes at silencing IL-2 production was demonstrated for all T-cell to exosome ratios, compared to A549 derived exosomes, in both static 2D disks and 3D scaffolds. Exosome vesicle heterogeneity has been observed for exosomes obtained from the same cell type [92], and this variability is expected to be more pronounced when obtaining exosomes from different cell lines.

Incorporating unidirectional flow in a co-culture of exosomes and T-cells attached to RGD-functionalized surfaces generates an environment where the effect of exosomes on T-cells is monitored under conditions that more closely mimic their physiological microenvironment. The exact flow rate of the perfused media was not selected with physiological conditions in mind, but we tested the ability of T-cells to withstand shear forces, which have been shown at high levels to cause T-cell detachment. One important component of the perfusion experiment is that the exosomes, being part of the cell culture media, do not reside in the immediate vicinity of the cultured T-cells, but instead circulate with the media and interact with the T-cells for short periods in repetitive fashion. In that sense, the effective interactions of exosomes in the immediate vicinity of T-cells are less frequent in perfusion cultures, and it is expected that higher exosome levels will be required to accomplish the same level of IL-2 production silencing than in static conditions. The 3D comparison showed that the IL-2 production decreases were lower than in 2D static cultures with the same T-cell to exosome ratios. As can be seen in Figure 8B, the T-cell to exosome ratio required to accomplish silencing of IL-2 production was 1:1000 with unidirectional flow, whereas when under static conditions, 1:20 was adequate to achieve the same outcome. Clearly, the results demonstrate a significant shift to higher level of exosomes in perfusion cultures compared to static ones. In a cancer exosome study, the blood of melanoma patients was collected, and within each mL of blood collected, the exosome profile was examined. It was found that of the 10^10^ exosomes in the blood found, the total percentage of cancer exosomes was 20% to 70% within these patients [93]. Comparing that number with the expected number of T-cells in a healthy individual, the number of cancerous exosomes is much higher than the number of T-cells, with T-cells comprising 20–50% of the 10^6^ to 10^7^ cells/mL in blood. The observations on cancerous exosomes enacting IL-2 silencing show that very few exosomes can accomplish that end under static conditions; however, when flow perfusion is added, the level of cancerous exosomes needed for silencing IL-2 production by T-cells returns to the vicinity of the levels of exosomes observed in cancerous patients. This fact denotes that the interactions of cancerous exosomes with T-cells under flow perfusion may better resemble the physiological interactions of T-cells with cancerous exosomes. Flow perfusion will be a valuable tool in future studies on the interactions of cancerous exosomes with T-cells [94]. The ability to incorporate pharmaceuticals in the culture media and study the potential pharmacokinetic effects on interactions between exosomes and surface-attached T-cells is an additional benefit of the proposed culture system.

## 5. Conclusions

A new methodology has been developed to study immobilized T-cells with cancer-exuded exosomes using 3D-printed, RGD-functionalized PLLA scaffolds with controllable RGD densities in a flow perfusion bioreactor. Using this novel experimental technique, we have identified the critical number of exosomes required to suppress the production of IL-2 by activated CD8+ T-cells in static and dynamic flow environments. We believe that the proposed system recapitulates the T-cell and exosome interactions in physiological conditions well, and could become a platform for studying the interactions of chemotherapeutics, cancer exosomes, and T-cells in vivo.

## Data Availability

The data presented are available from the corresponding author upon request.
